# Evaluation of the Conrad 30 Waiver Program’s Success in Attracting International Medical Graduates to Underserved Areas

**DOI:** 10.1001/jamahealthforum.2023.2021

**Published:** 2023-07-28

**Authors:** Tarun Ramesh, Sarah E. Brotherton, Gregory D. Wozniak, Hao Yu

**Affiliations:** 1 Department of Population Medicine, Harvard Medical School and Harvard Pilgrim Health Care Institute, Boston, Massachusetts; 2 American Medical Association, Chicago, Illinois

## Abstract

This cross-sectional study evaluated the growth and distribution of physicians in the Conrad 30 Waiver program during the past 2 decades.

## Introduction

Physician shortage is a persistent challenge facing the US.^1^ As part of the ongoing efforts to combat the shortage, the Conrad 30 Waiver program offers an important legal path for noncitizen international medical graduates (IMGs) to bolster the US physician workforce. Noncitizen IMGs can legally train in US medical residency programs through the H-1B and J-1 visa pathways, with the vast majority entering through J-1.^2^ The Immigration and Nationality Act requires that those trainees return to their country of origin for 2 years when they complete their training. During that time, they may apply for a traditional immigrant visa to return to the US. However, the Conrad program waives the requirement by allowing those trainees to convert their J-1 to H-1B nonimmigrant status in exchange for a 3-year full-time commitment to practice medicine in underserved areas.^3^

The Conrad program, authorized by the 1994 Immigration and Nationality Technical Corrections Act, is both the predominate pipeline for noncitizen IMGs with J-1 visas to enter the domestic workforce and the largest supplier of physicians to shortage areas.^4^ Since 2003, Congress has increased the waiver cap to 30 per state per year. However, there is little information on the growth and distribution of Conrad physicians during the past 2 decades. This study aimed to fill this evidence gap.

## Methods

This retrospective cross-sectional analysis of all US states and the District of Columbia used data from 2001 to 2020 from the Rural Recruitment and Retention Network. These data were publicly available and determined as not human participant research by the corresponding author’s institutional review board. Informed consent was waived because the data were deidentified. The study followed the Strengthening the Reporting of Observational Studies in Epidemiology (STROBE) reporting guideline. We assessed trends in the number of Conrad physicians and their distribution by specialty and geography. All tests were 2-sided, used an α level of .05, and were conducted in Stata statistical software, version 17.0 (StataCorp). The data were analyzed between May 30, 2022, and November 2, 2022.

## Results

**Figure 1.**
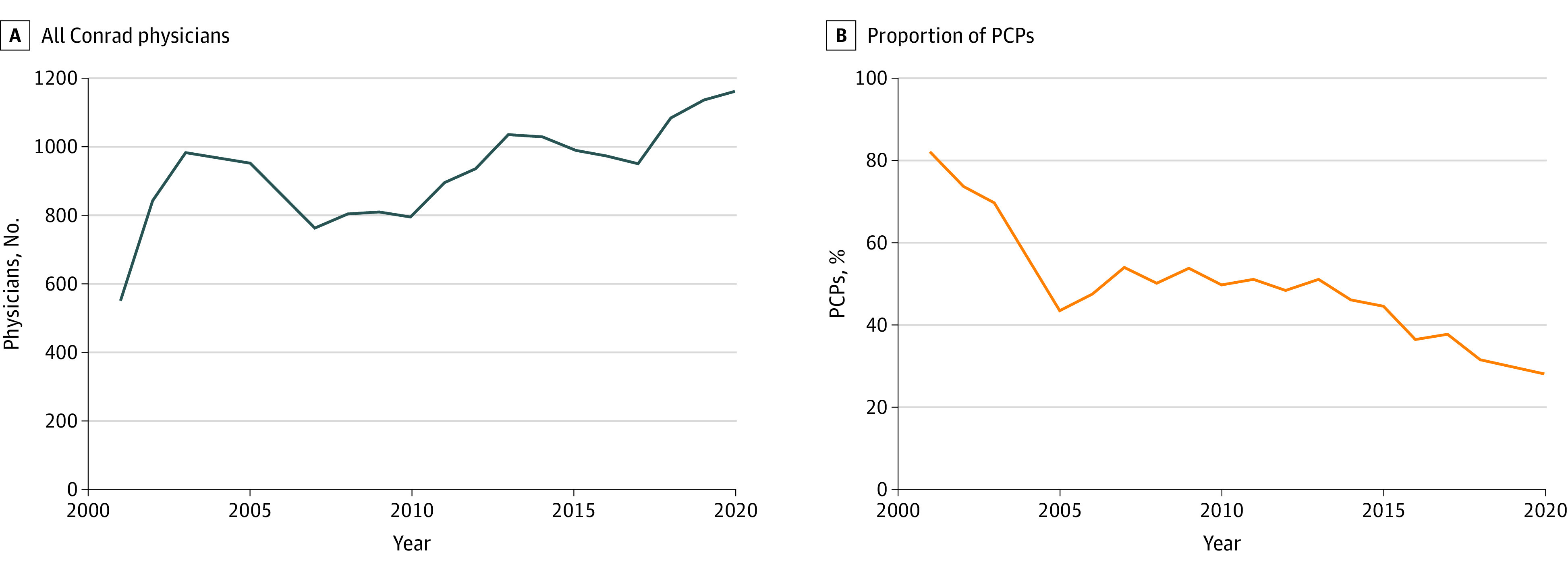
Number of Conrad Physicians and Proportion of Primary Care Physicians (PCPs) per Year From 2001 to 2020 A, The annual number of Conrad physicians increased from 550 in 2001 to 1162 in 2020, a 111% increase. B, The proportion of PCPs supported by the program decreased from 82% to 28% in the study period. The number of nonprimary care physicians grew 6-fold from 127 to 793 during the 2 decades.

The Conrad program recruited 18 504 physicians from 2001 to 2020. The annual number of Conrad physicians increased from 550 in 2001 to 1162 in 2020, a 111% increase, while the number of unfilled slots fell from 950 to 338, respectively (Figure 1).

**Figure 2.**
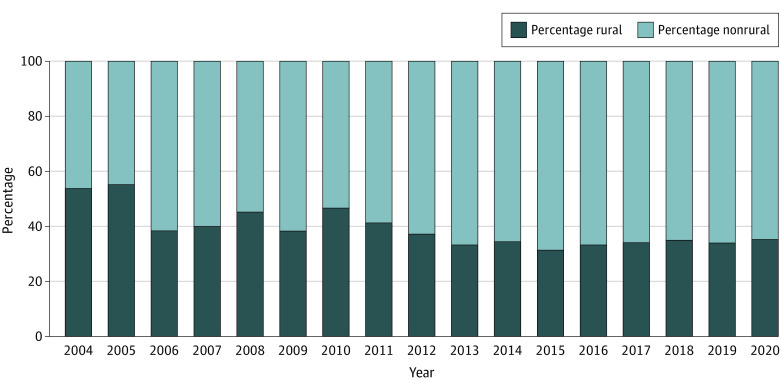
Percentage of Conrad Physicians Recruited to Rural Areas per Year From 2004 to 2020 In 2020, 34% of Conrad physicians served rural areas, decreasing from 49% in 2004.

Differential growth patterns were identified by specialty and geography. The proportion of primary care physicians (PCPs) supported by the program fell from 82% (n = 471) to 28% (n = 402) in the study period (Figure 1). The number of nonprimary care physicians grew 6-fold from 127 to 793 during the 2 decades. In 2020, 34% (n = 402) of Conrad physicians served rural areas, declining from 49% (n =471) in 2004 (Figure 2).

## Discussion

This retrospective cross-sectional study revealed 2 trends in the Conrad program during the past 2 decades: the declining proportions of recruited PCPs and rural physicians. The decreased proportion of Conrad PCPs is a substantial finding given the severe shortage of primary care clinicians in the nation, with more than 86 million (26%) Americans living in primary care Health Professional Shortage Areas (HPSAs) in 2021.^5^ However, considering the importance of specialty care physicians on health outcomes and continued shortages of those physicians, states should be encouraged to use the Conrad program to recruit those physicians to meet their needs.^6^ Declining Conrad physicians in rural areas suggests that the program may not have fulfilled its original goal of improving physician supply in HPSAs, of which the majority (65%) are in rural counties.

Although the present study assessed national trends of the Conrad program, we did not analyze state-level drivers of variations in program implementation. Further exploration of the Conrad program is needed, such as discussions with state officials about their definition of program success, their use of 10 additional FLEX slots, and their efforts to balance between PCPs and other specialists and between rural and urban areas. Identifying and sharing best practices across states can make the Conrad program more effective for improving physician supply in underserved areas.

## Notes

Supplement.
Data Sharing Statement
