# Supplementary material for: Evaluation of the Conrad 30 Waiver Program’s Success in Attracting International Medical Graduates to Underserved Areas
Source: JAMA Health Forum. 2023 Jul 28;4(7):e232021. doi: 10.1001/jamahealthforum.2023.2021 (PMC10383001; doi:10.1001/jamahealthforum.2023.2021)
Supplement: Supplement. — Data Sharing Statement [file jamahealthforum-e232021-s001.pdf]

# Data Sharing Statement

Ramesh. Evaluation of the Conrad 30 Waiver Program's Success in Attracting International Medical Graduates to Underserved Areas. *JAMA Health Forum*. Published July 28, 2023. doi:10.1001/jamahealthforum.2023.2021

**Data**
**Data available:** Yes
**Data types:** Other (please specify)
**Additional Information:** This study used secondary data, all of which are publicly available below.
**How to access data:** Data from the Rural Recruitment and Retention Network on physicians recruited by the Conrad 30 waiver program are available at: https://www.3rnet.org/Resources/J1-Waiver
**When available:** With publication

**Supporting Documents**
**Document types:** None

**Additional Information**
**Who can access the data:** Anyone requesting the data
**Types of analyses:** For research only.
**Mechanisms of data availability:** With investigator support
